# Headspace stir‐bar sorptive extraction combined with gas chromatography–mass spectrometry for trace analysis of volatile organic compounds in *Schisandra chinensis* Baillon (omija)

**DOI:** 10.1002/fsn3.3668

**Published:** 2023-09-07

**Authors:** Jae Hoon Lee, Yun‐Yeol Lee, Yun‐Sang Choi, Hae Won Jang

**Affiliations:** ^1^ Korea Food Research Institute Wanju South Korea; ^2^ Department of Food Science and Biotechnology Sungshin Women's University Seoul South Korea

**Keywords:** gas chromatography–mass spectrometry, headspace stir‐bar sorptive extraction, *Schisandra chinensis* Baillon, volatile organic compound

## Abstract

Analyzing volatile organic compounds (VOCs) in food is crucial but challenging. *Schisandra chinensis* Baillon (omija) is an herbal plant with various functional health activities. Previous VOC analyses focused on *S. chinensis* fruit but not its leaves. Therefore, VOCs in *S. chinensis* fruit and leaves were analyzed using headspace stir‐bar sorptive extraction (HS‐SBSE)‐GC–MS, and optimal conditions were established. Various factors, such as the sample preparation method, twister stir‐bar type, sample amount, extraction temperature, and extraction time, expected to affect extraction were carefully optimized. Under the optimal conditions, 35 and 40 VOCs were identified in *S. chinensis* fruit and leaves, respectively. This HS‐SBSE method is capable of rapid analysis and a low contamination rate without requiring organic solvents. These findings provide practical guidelines for HS‐SBSE applications in various food matrices by providing analytical methods for VOC detection.

## Graphical abstract

*Schisandra chinensis* (Turcz.) Baill., known as “omija” in Korea, is a well‐known medicinal crop. In previous studies, volatile compound analyses have focused on *S. chinensis* fruit only; however, the leaf is often used in tea, and analyses of volatile compounds in the leaves are lacking. Therefore, the purpose of this study was to develop and validate an optimized analytical method for the characterization and determination of volatile organic compounds in the fruit and leaves of *S. chinensis* using headspace stir‐bar sorptive extraction and GC–MS.

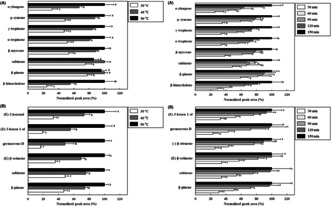

## 1 INTRODUCTION

*Schisandra chinensis* (Turcz.) Baill. is mainly cultivated in East Asian countries and is an herbal plant known as “omija” in Korea (Lee, Lee, et al., 2021). *Schisandra chinensis* is known as a medicinal crop, reportedly having various health functional activities, including antioxidant (Yang et al., 2019), antimicrobial (Teng & Lee, 2014), immunomodulatory (Dilshara et al., 2013), anti‐inflammatory (Lee, Lee, et al., 2021), antidepressant (Yan et al., 2016), anti‐cancer (Min et al., 2008), and anti‐obesity (Park et al., 2012) effects. *Schisandra chinensis* is widely used as a tea in East Asian countries such as China and South Korea (Li et al., 2018), and the tea has been reported to improve human body function and relieve fatigue. The analysis of volatile compounds in tea is very important (An et al., 2022; Xiao et al., 2022); however, in previous studies, volatile compound analyses have focused on *S. chinensis* fruit (Cheng et al., 2014; Park & Lee, 2021). Thus, volatile compound analyses of the leaf parts of *S. chinensis* are lacking. Flavor is one of the most important properties of food products and is an important factor in determining their acceptability and preference. Flavor is evaluated by considering several factors, such as aroma, taste, and other sensory attributes (Li et al., 2022). Among these factors, aroma, which is made up of volatile organic compounds (VOCs), has attracted attention in the field of food science (Song & Liu, 2018). One of the biggest reasons for this is that even trace amounts of certain VOCs in food can have a significant impact on the odor activity of food (Lee et al., 2019). However, it is challenging to accurately analyze VOC profiles in food because of their abundance and the fact that the concentrations of each VOC vary (Lee et al., 2019). Analysis of VOCs from samples is carried out in several steps, including sampling, sample extraction, separation, detection, and analysis (Marín‐San Román et al., 2020). Sample extraction is a very important step that can lead to serious errors owing to the loss of a large amount of analyte (Andrade‐Eiroa et al., 2016). Therefore, many sample extraction methods have been developed and are constantly being modified, and new methods have been developed to achieve optimal analytical results (Lee, Cha, et al., 2021; Marín‐San Román et al., 2020; Serrano de la Hoz et al., 2016). Liquid–liquid extraction, solid‐phase extraction, simultaneous distillation extraction, and distillation under reduced pressure, which are well‐known conventional sample extraction techniques, have been reported to have several problems, such as low reproducibility, low selectivity, and difficult automatization (Marín‐San Román et al., 2020; Song et al., 2021). Moreover, these conventional extraction methods use a large amount of solvent, which increases the risk of workplace and environmental pollution (Silvestre et al., 2009). Solid‐phase microextraction (SPME) and stir‐bar sorptive extraction (SBSE) have been developed as new extraction methods to solve these problems. Both methods eliminate the use of solvents and are relatively simple—extraction and concentration are performed in one step (Ochiai et al., 2018; Perestrelo et al., 2011). SBSE has a smaller sample loss volume than SPME and is more sensitive and robust than SPME (Lee et al., 2018; Prieto et al., 2010). According to previous reports, SBSE is 50–250 times more sensitive than SPME (Marín‐San Román et al., 2020). The SBSE technique uses a magnetic bar, called a “twister,” to extract the sample. This magnetic bar, which is coated with a polymeric extracting phase, extracts and enriches organic compounds from aqueous matrices (David & Sandra, 2007). Headspace‐SBSE (HS‐SBSE) is an analytical method that can be applied to all solid, liquid, and gaseous samples, in which the twister is introduced into a vial adapted for headspace (Marín‐San Román et al., 2020). Headspace sampling has the advantage of very high selectivity because only volatile and semi‐volatile organic compounds are released into the headspace. In addition, because there is no contact between the sample and the phase, both the background adsorption and matrix effect are reduced, and the life expectancy of the bar is increased (Grossi et al., 2008). To the best of our knowledge, only a few studies have identified VOCs in the fruit and leaves of *S. chinensis* using the HS‐SBSE extraction method. Therefore, this study aimed to develop and validate an optimized analytical method for the characterization and determination of VOCs in the fruit and leaves of *S. chinensis* using HS‐SBSE and GC–MS.

## 2 MATERIALS AND METHODS

### 2.1 Materials and reagents

To determine the sample pretreatment method to extract volatile compounds from the fruit and leaves of *S. chinensis*, raw fruit and leaf samples were freeze‐dried at −70°C for 53 h, and then the ratio of the raw samples to the retrieved ones was measured. The retrieved samples of equivalent weight to the raw samples were used as extraction samples. The amounts of retrieved fruit and leaf samples after freeze drying were 18.07% ± 0.17% and 17.16% ± 0.94% of the raw samples, respectively. The raw samples, blended raw samples, and freeze‐dried blended samples were placed in a Twister headspace vial (Gerstel) and used for the comparative analysis of the sample pretreatment methods to extract VOCs. The fruit and leaves of *S. chinensis* were obtained from Hyojongwon, a local producer located in Munkyung, Gyeosangbukdo, Korea. All *S. chinensis* samples were stored in a freezer at −20°C until analysis. Before HS‐SBSE, all samples were ground with dry ice to prevent the thermal loss of VOCs. All chemical reagents used in this study were purchased from Sigma‐Aldrich Corporation (St. Louis, MO, USA). An internal standard solution (phenethyl alcohol) was prepared by dilution in distilled water.

### 2.2 Optimization of HS‐SBSE

To establish the optimal conditions for analyzing VOCs in the fruit and leaves of *S. chinensis* using HS‐SBSE, different preparation methods, twister stir‐bar types, sample amounts, extraction temperatures, and extraction times were evaluated. The preparation methods for the analysis included raw samples, blended raw samples, and freeze‐dried blended samples. We tested two different stir bars, comprising either polydimethylsiloxane (PDMS) or ethylene glycol (EG) silicone. The tested sample amounts were 0.3, 0.6, and 0.9 g for fruit and 0.1, 0.2, 0.4, and 0.6 g for leaves. The tested temperatures were 30, 40, and 50°C, and the tested extraction times were 30, 60, 90, 120, and 150 min. Finally, analysis was performed using GC–MS under the conditions mentioned in the next section, and the optimal conditions were selected by comparing the adsorption efficiencies.

### 2.3 GC–MS analysis

For the extraction of *S. chinensis*, fruit (0.3 g) and leaves (0.4 g) were placed in Twister headspace vials (Gerstel) before 100 μL of phenethyl alcohol (10 g/L) solution was added as an internal standard. Phenethyl alcohol was chosen as the internal standard after confirming its absence in *S. chinensis* samples and its effective separation from other VOCs under optimized conditions. A Twister headspace insert (Gerstel) was placed in the upper part of the vial. A twister was then placed in the insert, and the vial was sealed with a crimp cap (Gerstel). For extraction, a vial containing a stir bar was placed in an agitator or a heating block. After extraction, the stir bar was placed in a Twister desorption liner (Gerstel) before the analytes on the stir bar were thermally desorbed in a thermal desorption unit. VOCs were identified in *S. chinensis* samples, together with their relative peak areas and RIs on DB‐WAX. The quantification of VOCs was performed using the peak area ratio (peak area of each compound/peak area of the internal standard). Due to the absence of standard curves for individual characteristic volatile compounds, semi‐quantitative determinations were carried out with phenethyl alcohol as the internal standard. While no standard curves were available, the use of relative concentrations remains valuable for the analysis of volatile profiles in foods (Jang et al., 2021; Lee et al., 2021; Xiao et al., 2014; Yao et al., 2015). Therefore, in this study, the relative contents of VOCs are expressed as equivalents of the internal standard (100 μL of 10 g/L phenethyl alcohol) in the HS‐SBSE‐GC–MS analysis. All experiments were performed in triplicate. The tentative identification of VOCs was performed by comparing the retention indices and mass spectra with those of the mass spectral library. The experimental retention index of VOCs in *S. chinensis* samples was calculated using saturated alkane standards containing C7–C40 (Sigma‐Aldrich, Steinheim, Germany), and the mass spectra of each VOC were compared to the information obtained from the Wiley and NIST 08 library databases. The extracted VOCs were analyzed using an HP 6980 gas chromatograph coupled with an HP 5973 mass‐selective detector (Agilent Technologies). The gas chromatograph was equipped with a 60‐m DB‐WAX column (internal diameter = 0.25 mm and thickness = 0.25 μm; Agilent Technologies). The temperature of the thermal desorption unit was programmed to increase from 50 to 220°C at 60°C/min for 5 min. The desorption flow in the thermal desorption unit was maintained at 50 mL/min in splitless mode. The temperature of the cooled injection system was maintained at −20°C with liquid nitrogen gas, and it was then programmed to 220°C at 12°C/s (held for 2 min) in 20:1 split mode, with a helium gas flow rate of 1.4 mL/min. The oven of the GC was maintained at 50°C (1 min) and then programmed to reach 210°C at 3°C/min. The transfer line, ion source, and quadrupole were maintained at 250, 230, and 150°C, respectively. The mass spectra were employed in full‐scan mode, and the mass range was collected between 35 and 400 m/z.

### 2.4 Statistical analysis

All experiments were performed in triplicate, and the data are presented as the mean ± SD. Data were statistically analyzed using one‐way analysis of variance (ANOVA) with Duncan's multiple‐range test (*p* < .05) and student's *t*‐test in SPSS Statistics 20 (SPSS Inc.).

## 3 RESULTS AND DISCUSSION

### 3.1 Optimization of HS‐SBSE extraction conditions

To establish the optimal conditions for the analysis of VOCs in *S. chinensis* fruit and leaves, the factors affecting extraction efficiency, including the sample preparation method, type of stir bar, sample amount, extraction temperature, and time, were established.

#### 3.1.1 Effect of sample preparation

**FIGURE 1:**
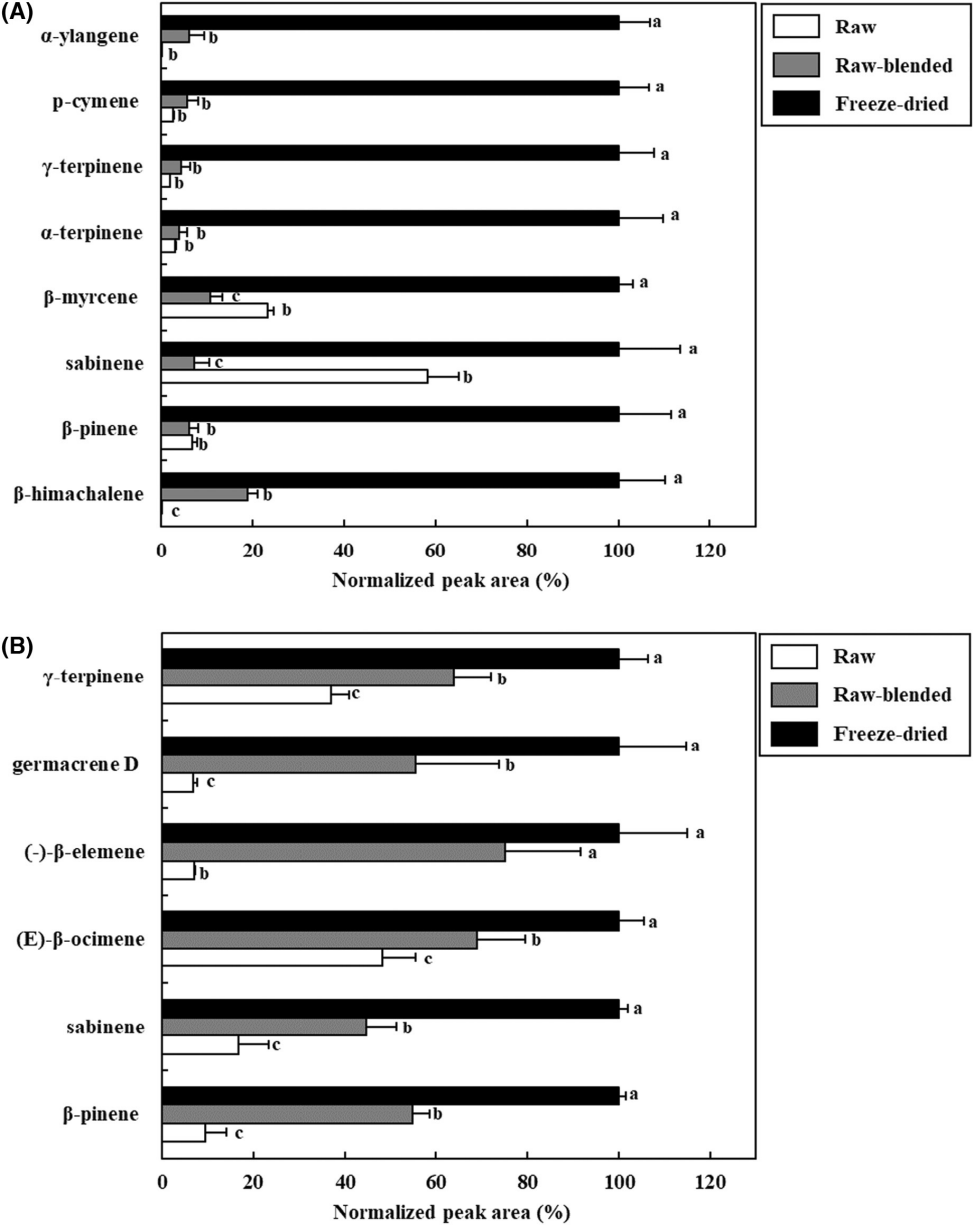
Effect of the sample preparation method using headspace stir‐bar sorptive extract‐GC–MS for the extraction of volatile organic compounds in *Schisandra chinensis* fruit (A) and leaves (B). Values are expressed as the mean ± SD. Different letters (a–c) among samples indicate significant differences calculated via one‐way ANOVA followed by Duncan's multiple‐range test (*p* < .05). Normalized peak area (%) = peak area of sample/the highest peak area of sample ×100.

To confirm the optimal conditions for the sample preparation method, three types of samples were prepared and analyzed: raw samples, blended raw samples, and freeze‐dried blended samples. As shown in Figure 1, eight VOCs (α‐ylangene, p‐cymene, γ‐terpinene, α‐terpinene, β‐myrcene, sabinene, β‐pinene, and β‐himachalene) were commonly detected as the major components of *S. chinensis* fruit samples prepared by the three methods. Similarly, it was confirmed that six major VOCs (γ‐terpinene, germacrene D, (‐)‐β‐elemene, (E)‐β‐ocimene, sabinene, and β‐pinene) were commonly detected in the leaf samples of *S. chinensis* prepared by the three methods. When the extraction efficiencies of the three preparation methods were compared based on the peak area of major VOCs, it was confirmed that the freeze‐dried blended sample had a high extraction efficiency for both fruit and leaves (*p* < .05). Thus, the freeze‐dried blended method was better suited for extraction efficiency.

#### 3.1.2 Effect of the twister stir‐bar type

**FIGURE 2:**
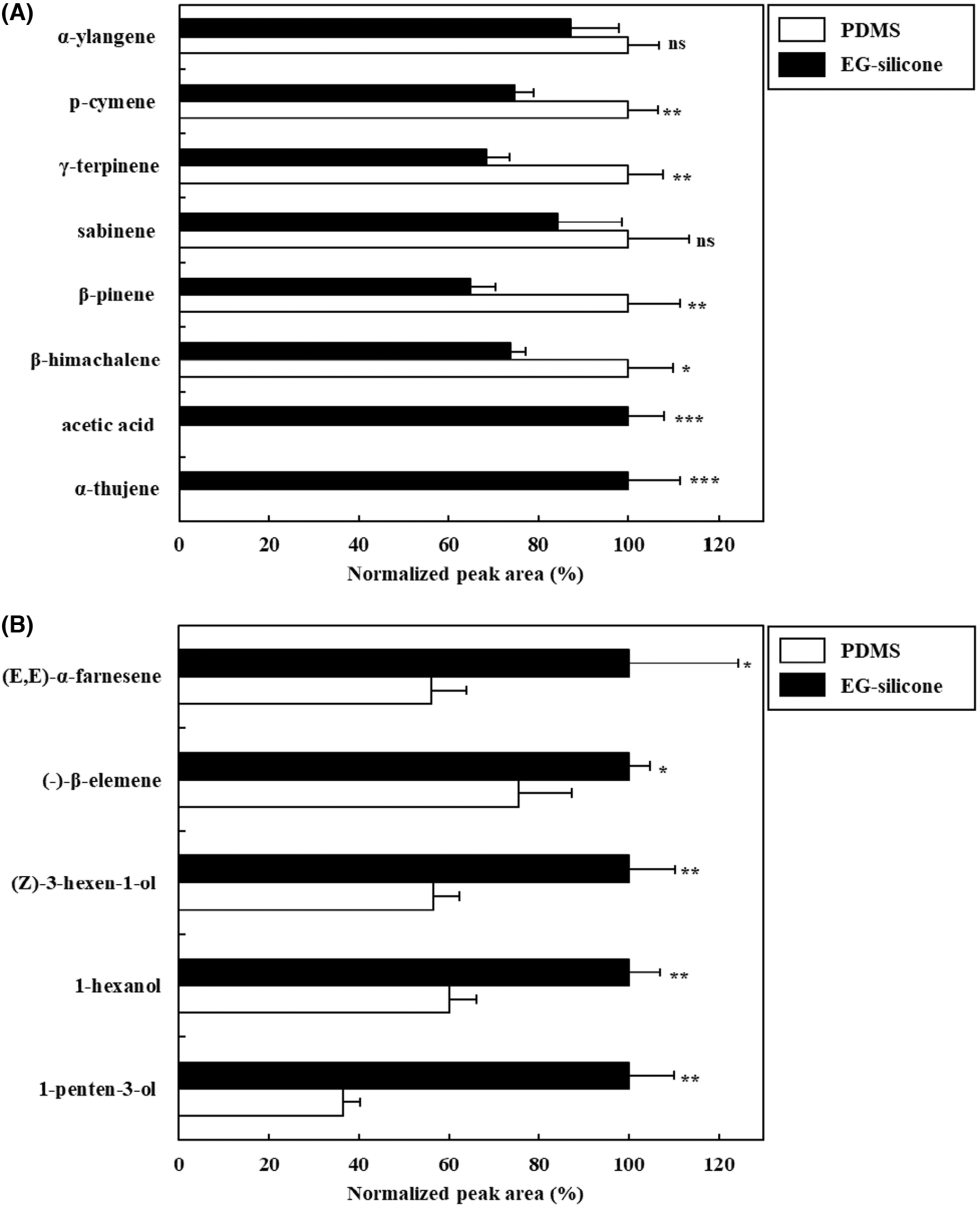
Effect of the twister stir bar using headspace stir‐bar sorptive extract‐GC–MS for the extraction of volatile organic compounds in *Schisandra chinensis* fruit (a) and leaves (b). Values are expressed as the mean ± SD. Statistical differences are indicated by *p < .05, **p < .01, and ***p < .001 (Student's *t*‐test) for comparisons between EG‐silicone and polydimethylsiloxane stir bars; ns: not significant. Normalized peak area (%) = peak area of sample/the highest peak area of sample ×100.

After choosing the freeze‐dried blended *S. chinensis* fruit and leaves in a headspace vial, extraction was performed using PDMS and EG‐silicone stir bars. In the fruit samples (Figure 2), major VOCs, such as α‐ylangene, p‐cymene, γ‐terpinene, sabinene, β‐pinene, and β‐himachalene, were detected with both the PDMS and EG‐silicone stir bars. When the EG‐silicone stir bar was used, the extraction efficiency of several VOCs was lower than that of PDMS one, but it was confirmed that acetic acid and α‐thujene were detected only with EG‐silicone. In contrast, in the leaf samples, major VOCs, including (E,E)‐α‐farnesene, (‐)‐β‐elemene, (Z)‐3‐hexen‐1‐ol, 1‐hexanol, and 1‐penten‐3‐ol, were detected with both stir bars. However, when the EG‐silicone stir bar was used, the extraction efficiency was higher based on the peak area (*p* < .05 or .01). This result is considered to be because EG has a higher affinity for polar substances than PDMS (Marín‐San Román et al., 2020). Thus, the use of an EG‐silicone stir bar for HS‐SBSE extraction of VOCs from *S. chinensis* fruit and leaves is recommended.

#### 3.1.3 Effect of sample amount

**FIGURE 3:**
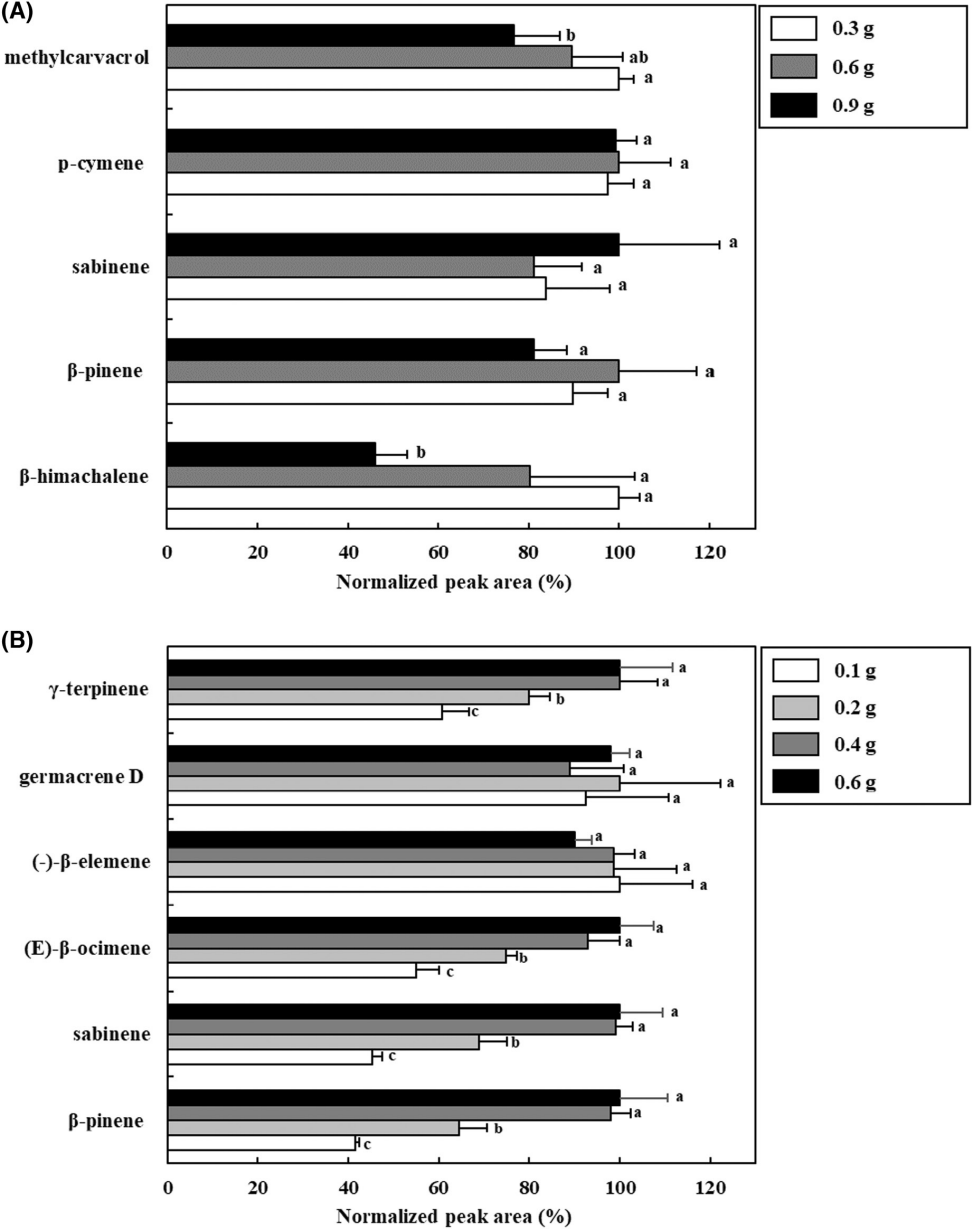
Effect of sample amount using headspace stir‐bar sorptive extract‐GC–MS for the extraction of volatile organic compounds in *Schisandra chinensis* fruit (A) and leaves (B). Values are expressed as the mean ± SD. Different letters (a–c) among samples indicate the significant differences calculated via one‐way analysis of variance followed by Duncan's multiple‐range test (*p* < .05). Normalized peak area (%) = peak area of sample/the highest peak area of sample ×100.

To optimize the sample weight of freeze‐dried blended *S. chinensis* fruit and leaves using HS‐SBSE, VOCs were extracted at 50°C for 2.5 h with an EG‐silicone stir bar. As shown in Figure 3, the extraction efficiencies of methyl carvacrol, p‐cymene, sabinene, β‐pinene, and β‐himachalene, which are the main VOCs of *S. chinensis* fruit, were compared by weight, and there was no significant difference (*p* > .05) in the extraction efficiency according to the weight of the sample. Therefore, 0.3 g was considered the most suitable for extracting *S. chinensis* fruit samples. In *S. chinensis* leaves, it was confirmed that the extraction efficiency increased significantly as the amount of sample increased from 0.1 to 0.4 g (*p* < .05) for the six main VOCs (γ‐terpinene, germacrene D, (‐)‐β‐elemene, (E)‐β‐ocimene, sabinene, and β‐pinene). However, there was no significant difference between 0.4 and 0.6 g of the sample (*p* > .05); thus, the optimum sample weight for analysis of VOCs from *S. chinensis* leaves was estimated to be 0.4 g.

#### 3.1.4 Effect of extraction temperature

**FIGURE 4:**
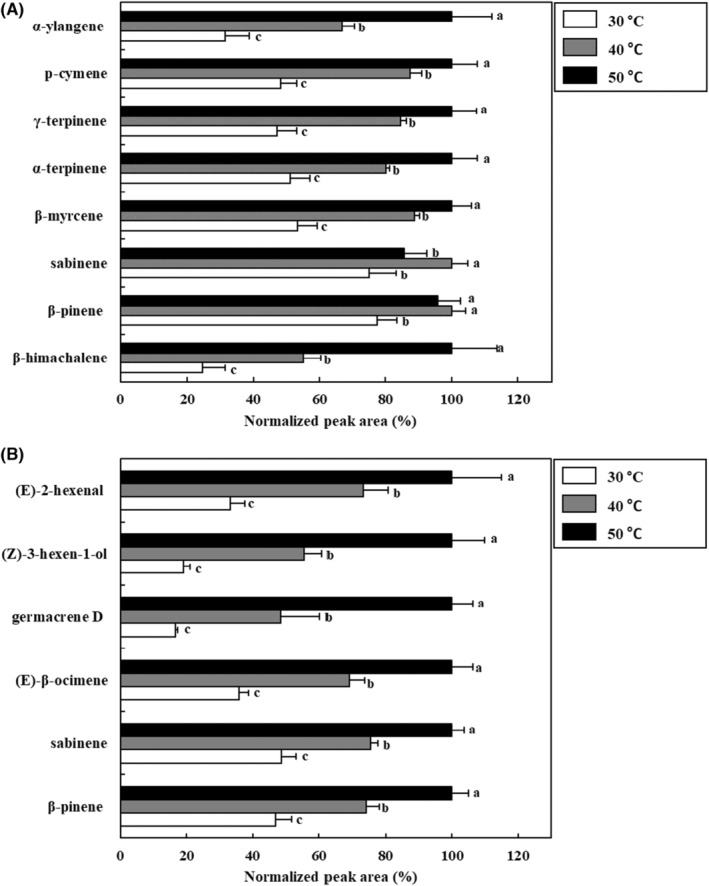
Effect of extraction temperature using headspace stir‐bar sorptive extract‐GC–MS for the extraction of volatile organic compounds in *Schisandra chinensis* fruit (A) and leaves (B). Values are expressed as the mean ± SD. Different letters (a–c) among samples indicate the significant differences calculated via one‐way analysis of variance followed by Duncan's multiple‐range test (*p* < .05). Normalized peak area (%) = peak area of sample/the highest peak area of sample ×100.

The optimal extraction temperature for obtaining the highest extraction efficiency of VOCs from *S. chinensis* fruit and leaves using HS‐SBSE was examined by performing extraction at 30, 40, and 50°C. As shown in Figure 4, the peak areas of the VOCs (α‐ylangene, p‐cymene, γ‐terpinene, α‐terpinene, β‐myrcene, sabinene, β‐pinene, and β‐himachalene) at 50°C were the highest among different temperatures in the case of *S. chinensis* fruit (*p* < .05). Similarly, the peak areas of the VOCs ((E)‐2‐hexenal, (Z)‐3‐hexen‐1‐ol, germacrene D, (E)‐β‐ocimene, sabinene, and β‐pinene) from *S. chinensis* leaves considerably increased until extraction reached 50°C. Therefore, to achieve a high extraction efficiency of *S. chinensis* fruit and leaves, the extraction temperature was set to 50°C.

#### 3.1.5 Effect of extraction time

**FIGURE 5:**
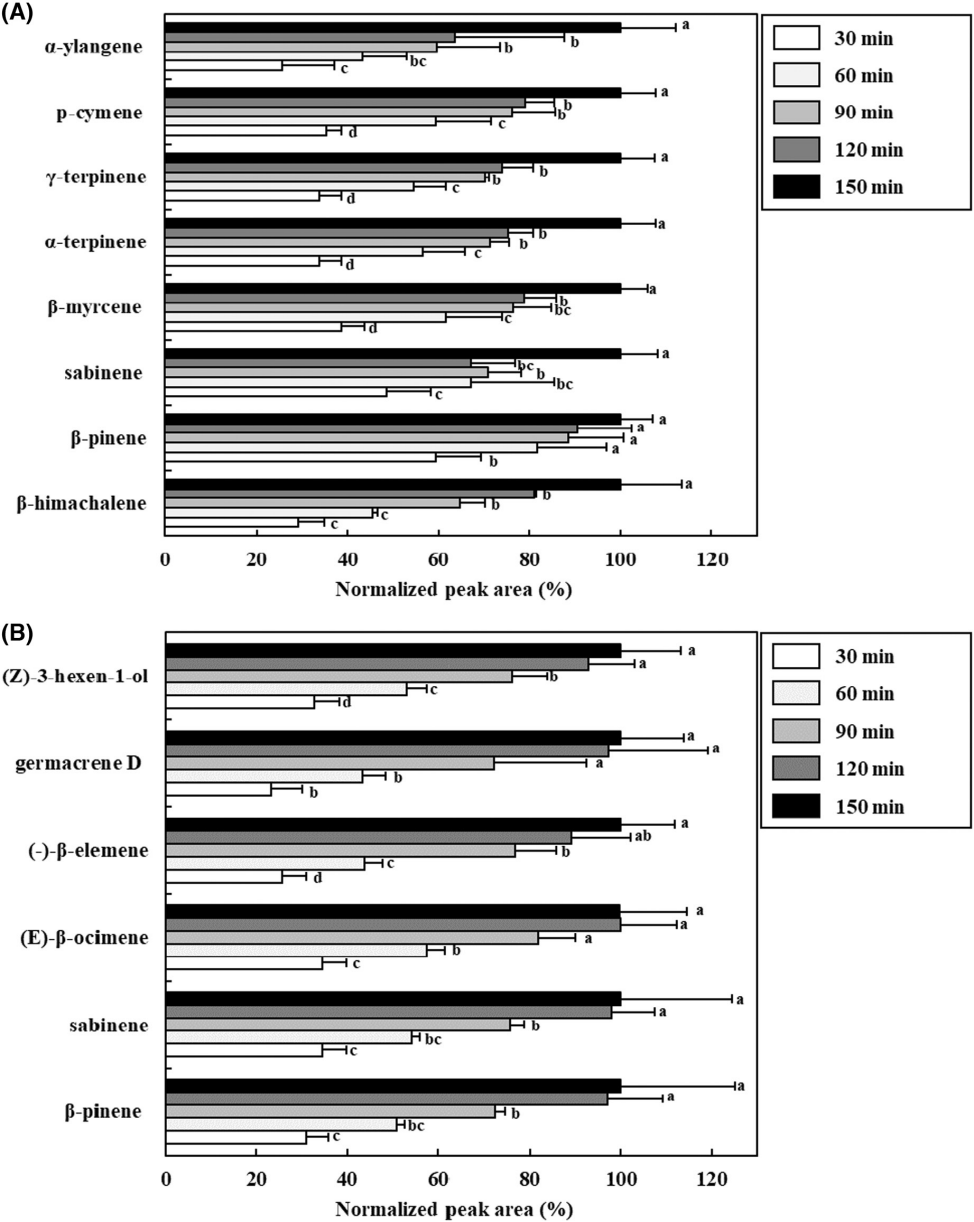
Effect of extraction time using headspace stir‐bar sorptive extract‐GC–MS for the extraction of volatile organic compounds in *Schisandra chinensis* fruit (A) and leaves (B). Values are expressed as the mean ± SD. Different letters (a–d) among samples indicate the significant differences calculated via one‐way analysis of variance followed by Duncan's multiple‐range test (*p* < .05). Normalized peak area (%) = peak area of sample/the highest peak area of sample ×100.

Extraction time is an important parameter that affects the extraction process of headspace sampling (Zhao et al., 2009). The optimal extraction time for obtaining the highest extraction efficiency was confirmed by performing extraction for 30, 60, 90, 120, and 150 min. As shown in Figure 5, in the case of *S. chinensis* fruit, the eight major VOCs (α‐ylangene, p‐cymene, γ‐terpinene, α‐terpinene, β‐myrcene, sabinene, β‐pinene, and β‐himachalene) were detected at the highest levels after 150 min of extraction (*p* < .05). In contrast, in the case of *S. chinensis* leaves, the extraction efficiency of six VOCs ((Z)‐3‐hexen‐1‐ol, germacrene D, (‐)‐β‐elemene, (E)‐β‐ocimene, sabinene, and β‐pinene) was examined, and it was confirmed that the extraction efficiency significantly increased up to 120 min (*p* < .05). However, there was no significant difference between the 120 min and 150 min extraction times (*p* > .05). Thus, the optimal extraction times for *S. chinensis* fruit and leaves were set to 150 and 120 min, respectively.

### 3.2 Qualitative and quantitative analyses

#### 3.2.1 Selection of the internal standard

To evaluate the internal standard for quantifying the VOCs of *S. chinensis* using HS‐SBSE, eight internal standards, including phenethyl alcohol, methyl octanoate, tetradecane, ethyl nonanoate, 1‐hexanol, hexyl butanoate, hexyl 2‐methyl butanoate, hexyl isobutyrate, and hexyl hexanoate, were tested. As a result, the peaks of seven internal standards, except for phenethyl alcohol, tended to overlap with those of VOCs extracted from *S. chinensis* fruit and leaves or affected the total peak area. In addition, an internal standard with high water solubility was prioritized to take advantage of the environmentally friendly HS‐SBSE, which does not use a solvent. Therefore, phenethyl alcohol was selected as the internal standard for the qualitative and quantitative analyses in this study.

#### 3.2.2 Analysis of VOCs in *S. chinensis* fruit and leaves using HS‐SBSE under optimal conditions

The optimal conditions for analyzing VOCs in *S. chinensis* fruit and leaves by HS‐SBSE were as follows: sample preparation method = freeze‐dried blended; twister stir‐bar type = EG‐silicone stir bar; sample amount = 0.3 g (fruit) and 0.4 g (leaves); extraction temperature = 50°C; and extraction time = 150 min (fruit) and 120 min (leaves). Under the optimized extraction conditions, the volatile compounds in *S. chinensis* samples were evaluated using HS‐SBSE. In total, 56 VOCs were identified in all *S. chinensis* samples (Table 1).

**TABLE 1:** Volatile organic compounds of fruit and leaves of *Schisandra chinensis* obtained using headspace stir‐bar sorptive extract‐GC‐MS.

Peak No.	RT (min)	RI	Volatile organic compounds^a^	Relative concentration (mg/kg)^b^		Odor description^c^
				Fruit	Leaves	
1	7.03	884	Ethyl acetate	21.7 ± 1.4	‐	Grape, pineapple
2	7.46	914	2‐methylbutanal	‐	1.8 ± 0.3	Fermented, hazelnut
3	7.51	916	3‐methylbutanal	‐	1.9 ± 0.6	Acrid
4	8.63	977	Pentanal	‐	2.5 ± 0.2	Almond, malt
5	9.63	1022	α‐pinene	1327.6 ± 39.5	158.8 ± 27.0	Cedarwood, pine
6	9.70	1024	α‐thujene	668.6 ± 38.2	37.6 ± 5.2	Green, herb
7	10.82	1066	Camphene	975.5 ± 33.6	10.5 ± 1.5	Camphor, mothball
8	11.26	1082	Hexanal	‐	19.7 ± 2.9	Grass
9	12.16	1112	β‐pinene	1565.4 ± 43.8	101.4 ± 16.4	Pine, resin
10	12.53	1123	Sabinene	610.2 ± 46.0	359.2 ± 57.3	Turpentine, wood
11	13.04	1137	1,4‐dimethyl‐benzene (p‐xylene)	29.3 ± 1.0	‐	Metal
12	13.51	1151	3‐carene	490.8 ± 23.7	12.5 ± 1.9	Lemon, resin
13	13.70	1156	1‐penten‐3‐ol	‐	12.2 ± 2.9	Grass, green
14	14.07	1167	β‐myrcene	3683.5 ± 168.6	2067.8 ± 316.2	Geranium, must
15	14.13	1169	α‐phellandrene	849.4 ± 337.8	‐	Citrus, mint
16	14.69	1185	α‐terpinene	2155.9 ± 105.9	18.4 ± 2.7	Berry, lemon
17	15.41	1205	Limonene	1891.6 ± 98.9	60.7 ± 9.4	Lemon, orange
18	15.76	1214	β‐phellandrene	493.3 ± 26.6	18.3 ± 2.6	Mint, turpentine
19	15.84	1216	1,8‐cineole	261.7 ± 8.6	12.5 ± 2.2	Mint, sweet
20	16.10	1223	(E)‐2‐hexenal	‐	178.1 ± 25.7	Green, leaf
21	16.64	1237	(Z)‐β‐ocimene	249.2 ± 17.2	198.7 ± 27.9	Herb, flower
22	17.17	1250	γ‐terpinene	9218.7 ± 511.2	84.1 ± 13.7	Gasoline
23	17.39	1256	(E)‐β‐ocimene	‐	975.4 ± 145.6	Sweet, herb
24	18.23	1277	p‐cymene	3149.7 ± 168.0	31.1 ± 4.5	Solvent, citrus
25	18.73	1290	Terpinolene	798.7 ± 43.3	15.3 ± 2.3	Pine, plastic
26	19.94	1320	(Z)‐3‐hexenyl acetate	‐	1.3 ± 0.3	Green, banana
27	20.80	1341	6‐methyl‐5‐hepten‐2‐one	15.2 ± 0.5	6.4 ± 0.8	Strawberry
28	21.43	1356	1‐hexanol	‐	2.0 ± 0.3	Grass
29	22.77	1389	(Z)‐3‐hexen‐1‐ol	‐	44.9 ± 5.9	Bell pepper, green leaf
30	22.97	1394	2‐nonanone	113.4 ± 7.3	‐	Fragrant, fruit
31	24.97	1443	1‐isopropenyl‐4‐methylbenzene	39.5 ± 2.8	‐	Citrus, fresh
32	25.58	1457	Acetic acid	25.5 ± 2.8	‐	Sour
33	26.39	1477	δ‐elemene	‐	8.7 ± 0.8	Sweet, wood
34	27.28	1499	α‐ylangene	9116.3 ± 1031.1	‐	Fruit
35	27.38	1502	α‐copaene	126.4 ± 8.4	20.9 ± 2.7	Wood, spice
36	28.27	1523	α‐bourbonene	‐	1.9 ± 0.4	
37	28.49	1529	β‐bourbonene	‐	24.1 ± 3.6	Herb
38	28.54	1530	Benzaldehyde	163.0 ± 9.0	‐	Almond, burnt sugar
39	29.22	1547	β‐cubebene	28.2 ± 4.0	6.2 ± 1.7	Citrus, fruit
40	31.26	1597	(‐)‐β‐elemene	‐	183.7 ± 16.3	Sweet
41	31.29	1598	4‐isopropyl‐2‐methoxy‐1‐methyl‐benzene	1643.6 ± 165.3	‐	Carrot, celery
42	31.46	1602	2‐undecanone	96.8 ± 11.8	‐	Pineapple, rose
43	31.66	1608	β‐caryophyllene	‐	18.0 ± 2.8	Wood, spice
44	31.77	1610	4‐terpineol	346.0 ± 30.4	‐	Must, nutmeg
45	34.45	1682	α‐humulene	‐	16.3 ± 4.5	Balsamic, hop
46	35.08	1699	γ‐curcumene	65.0 ± 10.2	‐	
47	35.11	1700	α‐muurolene	‐	5.7 ± 1.2	Wood
48	35.92	1722	Germacrene D	‐	347.4 ± 82.3	Wood
49	36.45	1736	(‐)‐β‐bisabolene	314.1 ± 62.0	‐	Balsamic
50	36.49	1737	α‐selinene	‐	6.2 ± 0.6	Wood
51	37.07	1751	β‐himachalene	2856.5 ± 484.6	‐	Resin
52	37.07	1753	(E,E)‐α‐farnesene	‐	14.4 ± 4.1	Boiled vegetable
53	37.64	1769	(+)‐δ‐cadinene	118.8 ± 15.6	8.9 ± 2.7	Thyme, herb
54	37.78	1773	γ‐cadinene	‐	11.1 ± 1.5	Wood
55	38.02	1779	β‐sesquiphellandrene	27.8 ± 3.6	‐	Fruit, herb
56	39.35	1817	2‐tridecanone	84.4 ± 16.9	‐	Old nut

*Note*: All values are represented by the mean ± SD of three replicates. RI on DB‐WAX.

Abbreviations: ‐, not detected; RI, retention index; RT, retention time.

^a^ Identification of VOCs from the acquired spectra was achieved based on a high match factor (>800).

^b^ Relative concentration (mg/kg) was obtained by the following formula: Sample concentration = Internal standard concentration (mg/kg) × Sample peak area/Internal standard peak area.

^c^ Odor description from: https://www.vcf‐online.nl/VcfHome.cfm.

A total of 35 VOCs were identified from the extracts of *S. chinensis* fruit: 15 monoterpene hydrocarbons, 8 sesquiterpene hydrocarbons, 2 oxygenated terpenes, 4 ketones, 2 hydrocarbons, 1 ether, 1 ester, 1 aldehyde, and 1 acid compound. Quantitatively, the main volatile components of *S. chinensis* fruit were monoterpenes and sesquiterpenes, including β‐myrcene, α‐terpinene, limonene, γ‐terpinene, p‐cymene, α‐ylangene, and β‐himachalene; these findings are similar to those of a previous study (Lee et al., 2011). Kim et al. (2008) also reported that β‐myrcene, α‐terpinene, limonene, γ‐terpinene, and p‐cymene are the major compounds in the fruit of *S. chinensis*. γ‐Terpinene, a major compound in fruit, is known to effectively inhibit lipid oxidation, which is the main component of cell membranes (Guo et al., 2021). In addition, the biofunctional activities of β‐myrcene, which is present in the fruit and leaves, such as antioxidant and antibacterial activity, have been reported (Wang et al., 2019). According to a previous study (Wang et al., 2019), β‐myrcene, along with limonene, another major compound in fruit, has antibacterial activity against various foodborne pathogenic bacteria, including *Escherichia coli*, *Salmonella enterica*, and *Staphylococcus aureus*. In addition, the antibacterial and antioxidant activities of essential oils obtained from *S. chinensis* seeds have been reported, and the main components of the essential oils are α‐ylangene and β‐himachalene (Teng & Lee, 2014). These were the major compounds found in the fruit of *S. chinensis*.

The following 40 VOCs were identified from the extracts of *S. chinensis* leaves: 15 monoterpene hydrocarbons, 14 sesquiterpene hydrocarbons, 1 oxygenated terpene, 1 ketone, 1 ester, 5 aldehydes, and 3 alcohol compounds. Quantitatively, the main volatile components in *S. chinensis* leaves included sabinene, β‐myrcene, (E)‐β‐ocimene, (‐)‐β‐elemene, and germacrene D, similar to those found in a previous study (Zheng et al., 2005). Among the major compounds of *S. chinensis* leaves, germacrene D is also found in the leaves of various other plants and is characterized by its excellent antioxidant activity (Andrade‐Eiroa et al., 2016; Xie et al., 2015). In addition, (E)‐β‐ocimene is found in the leaves and stems of *Gypsophila bicolor*, and it has been reported to have antibacterial activity against Gram‐positive and Gram‐negative bacteria (Shafaghat & Shafaghatlonbar, 2011). In summary, the major compounds detected in the fruit and leaves of *S. chinensis* showed excellent biofunctional activity as well as aromatic components, suggesting that they are highly likely to be used in the food industry.

A total of 35 VOCs were detected in *S. chinensis* fruit, and 40 VOCs were detected in leaves, confirming that more VOCs were detected in the leaves. In addition, 19 VOCs were simultaneously detected in both the fruit and leaves. While the total concentration of VOCs was higher in the fruit, the leaves had 21 types of VOCs that were not found in the fruit. The leaf‐specific VOCs included (E)‐β‐ocimene, δ‐elemene, and (‐)‐β‐elemene, which were characterized by a pleasant odor.

Until now, the SBSE method has mainly been used to analyze liquids, such as beer, wine, juice, and milk. The use of the SBSE method for the analysis of solid foods, such as fruit and vegetables, is only possible after extraction with an organic solvent. However, in this study, the VOCs of solids (fruit and leaves) were successfully analyzed without using organic solvents through the HS‐SBSE method. The optimal conditions for analyzing VOCs in *S. chinensis* fruit and leaves using HS‐SBSE were confirmed through our experiments, and under these conditions, 35 and 40 VOCs were identified from *S. chinensis* fruit and leaf extracts, respectively. Thus, an analysis method with excellent reproducibility that reduces the number of by‐products and the rate of contamination of the sample was established in this study; furthermore, the method is fast and does not require organic solvents. These results support the use of HS‐SBSE for the analysis of VOCs in solid foods and are expected to improve food research in the future.

## AUTHOR CONTRIBUTIONS

**Jae Hoon Lee:** Data curation (equal); methodology (equal); visualization (equal); writing – original draft (equal). **Yun‐Yeol Lee:** Data curation (supporting); formal analysis (lead); methodology (equal). **Yun‐Sang Choi:** Conceptualization (supporting); resources (supporting); visualization (supporting). **Hae Won Jang:** Conceptualization (lead); funding acquisition (lead); investigation (lead); project administration (lead); resources (lead); supervision (lead); writing – review and editing (lead).

## CONFLICT OF INTEREST STATEMENT

The authors declare no conflict of interest.

## ETHICS STATEMENT

This study does not involve any human or animal testing.

## Data Availability

The data supporting the findings of this study are available from the corresponding author upon reasonable request.
